# Wrong-Way Eye Deviation During Eye Opening

**DOI:** 10.7759/cureus.84495

**Published:** 2025-05-20

**Authors:** Wataru Shiraishi, Yusuke Nakazawa, Yukiko Inamori, Yasutaka Iwanaga, Akifumi Yamamoto

**Affiliations:** 1 Department of Neurology, Kokura Memorial Hospital, Kitakyushu, JPN; 2 Department of Internal Medicine, Shiraishi Internal Medicine Clinic, Nogata, JPN; 3 Department of Neurology, Japan Community Healthcare Organization Kyushu Hospital, Kitakyushu, JPN; 4 Department of Neurology, Brain and Nerve Center, Fukuoka Central Hospital, Fukuoka, JPN; 5 Department of Neurology, National Hospital Organization Omuta National Hospital, Omuta, JPN

**Keywords:** contralateral eye deviation, eye deviation, large vessel stroke, smoking tobacco, wrong-way eye deviation

## Abstract

In most cases, eye deviation caused by brain lesions points toward the side of the lesion in supratentorial strokes and away from the lesion in infratentorial strokes. However, in rare cases of supratentorial lesions, the eyes may deviate to the opposite side, a condition known as "wrong-way eye deviation." A woman in her 60s with a history of untreated hypertension and heavy smoking was found unconscious at home and brought to the emergency department. On arrival, she was comatose, with a Glasgow Coma Scale score of 4 (E1V1M2), complete right-sided paralysis, and rightward eye deviation. Notably, her eyes remained in the midline when closed but deviated abruptly to the right when forcibly opened. Imaging revealed a large infarction in the left cerebral hemisphere due to occlusion of the left internal carotid artery, though collateral circulation preserved flow to the left middle cerebral artery. Electroencephalography showed no epileptic discharges, and antiseizure medications had no effect. A diagnosis of atherothrombotic cerebral infarction was made, and medical treatment was initiated. The patient’s level of consciousness gradually improved, but the unusual eye deviation persisted for nearly three weeks. This presentation is consistent with “wrong-way eye deviation,” a rare finding where the eyes deviate away from the side of a supratentorial lesion. What made this case distinct was the appearance of this deviation only during eye opening. This suggests selective impairment of the anterior voluntary eye movement pathway with relative preservation of the posterior automatic pathway. Further case accumulation is essential to better understand this rare phenomenon.

## Introduction

Eye deviation in patients with cerebral lesions typically occurs toward the lesion in supratentorial cases and away from the lesion in infratentorial cases [1-3]. However, in some cases of supratentorial lesions, eye deviation toward the contralateral side of the lesion can occur, a phenomenon referred to as “wrong-way eye deviation” [4,5]. Although the detailed mechanism of wrong-way eye deviation remains unclear, it has been observed in cases of thalamic hemorrhage or extensive hemispheric infarction and is considered a poor prognostic sign [6]. Johkura et al. reported that wrong-way eye deviation is caused by brainstem compression occurring from extended supratentorial lesions [4]; however, other reports mentioned that the mechanism of wrong-way eye deviation belongs to the abnormality of the smooth pursuit pathway [5]. Normally, eye movements are controlled by signals originating in the frontal eye fields of the cerebral cortex, transmitted through the brainstem gaze centers and cranial nerve nuclei, and ultimately reaching the extraocular muscles via cranial nerves III, IV, and VI [1]. Here, we present a unique case of wrong-way eye deviation associated with a massive supratentorial ischemic lesion. Our case presented two characteristic findings. First, our case showed wrong-way eye deviation when the eyes were opened, and the eye was in the midline when the eyes were closed. Second, the patient showed no brainstem compression during the disease course. As far as we searched, there are no similar cases, and we hypothesize that this unusual eye movement may have been caused by an imbalance between the two pathways of horizontal impulsive eye movements, resulting in wrong-way eye deviation during eye opening.

## Case presentation

**Figure 1:**
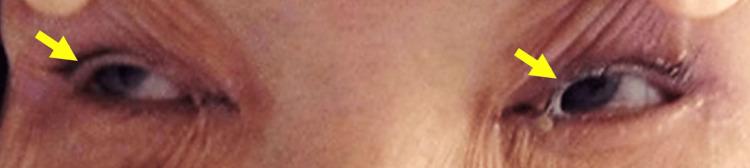
Photograph of the eye deviation to the right (the paralyzed side) during forced eye opening

The patient was a Japanese woman in her 60s. She had untreated hypertension and a smoking habit of two packs of cigarettes daily for over 40 years (Brinkman index: 800). One day, her family, who lived in the neighborhood, noticed she had not come to work and visited her home. She was found unresponsive on the floor and was transported to our hospital. Her blood pressure was 202/92 mmHg. Her pulse rate was 88 beats/minute and was regular. Neurological findings were as follows: she was unconscious with a Glasgow Coma Scale (GCS) of 4/15 (eye opening: E 1, verbal response: V 1, motor response: M 2). Her eyes were closed, and she showed minimal spontaneous movements. The pupils were 3.5 mm in diameter, and the light reflexes were normal. Her eyes impulsively deviated to the right on the forced eye opening (Figure 1).

There was no nystagmus. The right side of the body showed flaccid paralysis, and Babinski and Chaddock reflexes were positive in the right lower limb. The left side of her body showed occasional spontaneous movements. Her National Institutes of Health Stroke Scale (NIHSS) was 26. Blood examinations revealed only mild dyslipidemia and no elevation of D-dimer or brain natriuretic peptide (Table 1).

**Table 1:** Blood test results AST: aspartate aminotransferase, ALT: alanine aminotransferase, ALP: alkaline phosphatase, BUN: blood urea nitrogen, T-Chol: total cholesterol, LDL-C: low-density lipoprotein cholesterol, HDL-C: high-density lipoprotein cholesterol, CRP: C-reactive protein, HbA1c: hemoglobin A1c, BNP: brain natriuretic peptide, PT-INR: prothrombin time international normalized ratio, APTT: activated partial thromboplastin time, MPO-ANCA: myeloperoxidase antineutrophil cytoplasmic antibody, PR3-ANCA: proteinase-3-anti-neutrophil cytoplasmic antibodies

Parameter	Results	Reference data
Hemoglobin (g/dL)	14.9	13-17.9
White blood cell (/μL)	8,800	3,000-8,900
Platelet (×10^4^/μL)	26.8	12-39
AST (U/L)	19.1	13-30
ALT (U/L)	8	7-23
ALP (U/L)	434	106-322
Total protein (g/dL)	7	6.5-8.5
BUN (mg/dL)	15	8-10
Creatinine (mg/dL)	0.63	0-1.2
T-Chol (mg/dL)	276	130-219
LDL-C (mg/dL)	167	70-139
HDL-C (mg/dL)	57	40-80
Triglycerides (mg/dL)	147	30-149
Creatine kinase (U/L)	47	30-180
Sodium (mEq/L)	139	137-146
Potassium (mEq/L)	4.3	3.6-4.9
CRP (mg/dL)	1.14	<0.5
HbA1c (%)	5.5	4.6-6.2
BNP (pg/mL)	16.5	<18.4
PT-INR	1.03	0.9-1.1
APTT (seconds)	32.7	24-34
D-dimer (µg/mL)	0.4	<1
Antinuclear antibody	-	-
Anti-SS-A antibody	-	-
MPO-ANCA	-	-
PR3-ANCA	-	-

**Figure 2:**
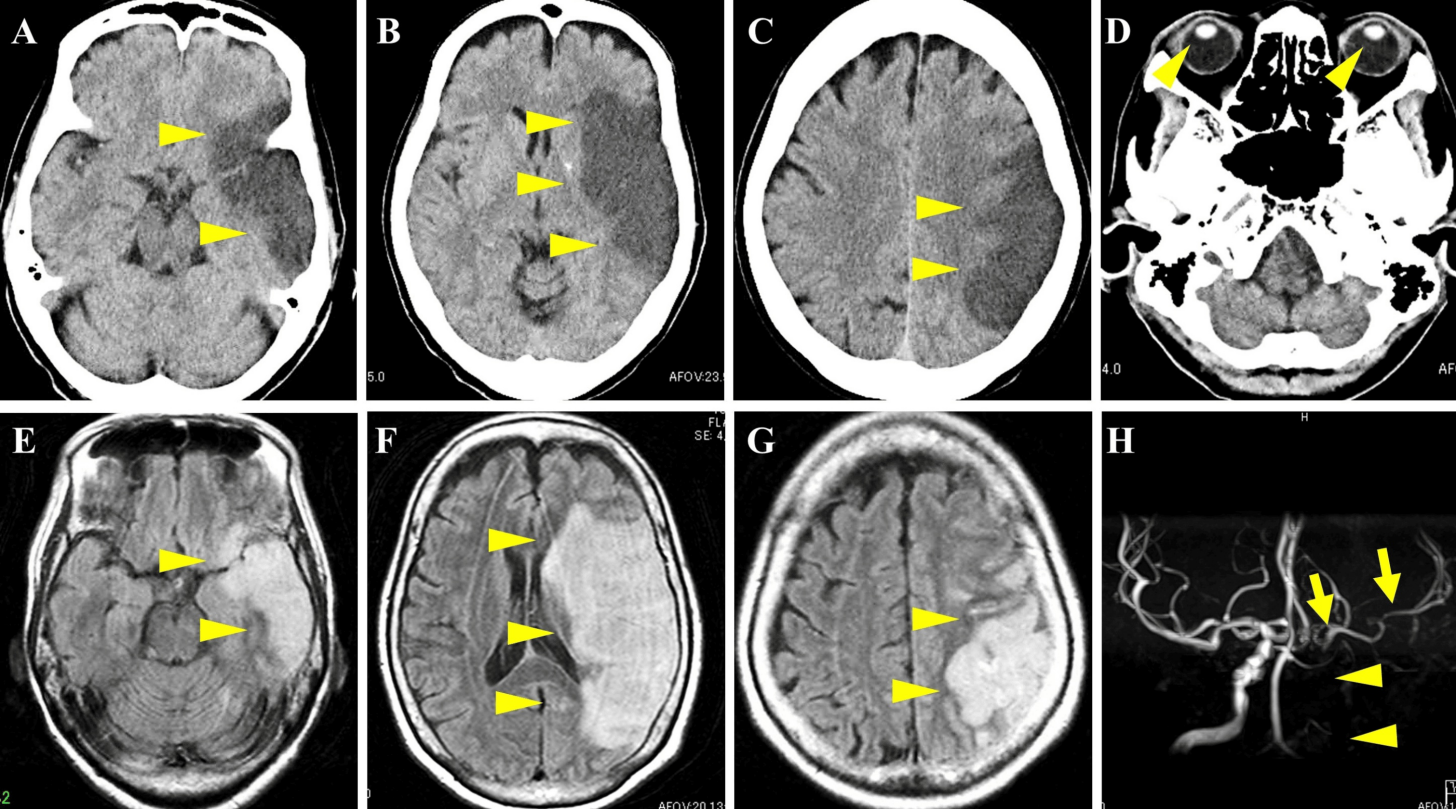
CT, MRI, and MRA of the brain CT (A-C) and MRI (E-G) showed extensive infarction of the left middle cerebral artery (arrowheads) without brain edema or herniation. The midline shift was minimal, and minor basal ganglia calcification was noted (B). MRA showed occlusion of the left internal carotid artery (D; arrowheads), but the left middle cerebral artery is faintly apparent from crossflow (H; arrows). Note that the frontal eye field (Brodmann’s area 8) and visual cortex (Brodmann’s area 39) were intact CT: computed tomography, MRI: magnetic resonance imaging, MRA: magnetic resonance angiography

Her head computed tomography (CT) scan showed a large low-density lesion in the middle cerebral artery region of the left cerebral hemisphere, with minimal cerebral edema and no hemorrhage (Figures 2A-2C). During the head CT scan, the eyes were closed, and the eye position was in the midline (Figure 2D). Brain magnetic resonance imaging also revealed acute brain infarction of the left hemisphere (Figures 2E-2G). The magnetic resonance angiography showed occlusion of the left internal carotid artery, but the left middle cerebral artery was faintly visualized from the crossflow (Figure 2H).

**Figure 3:**
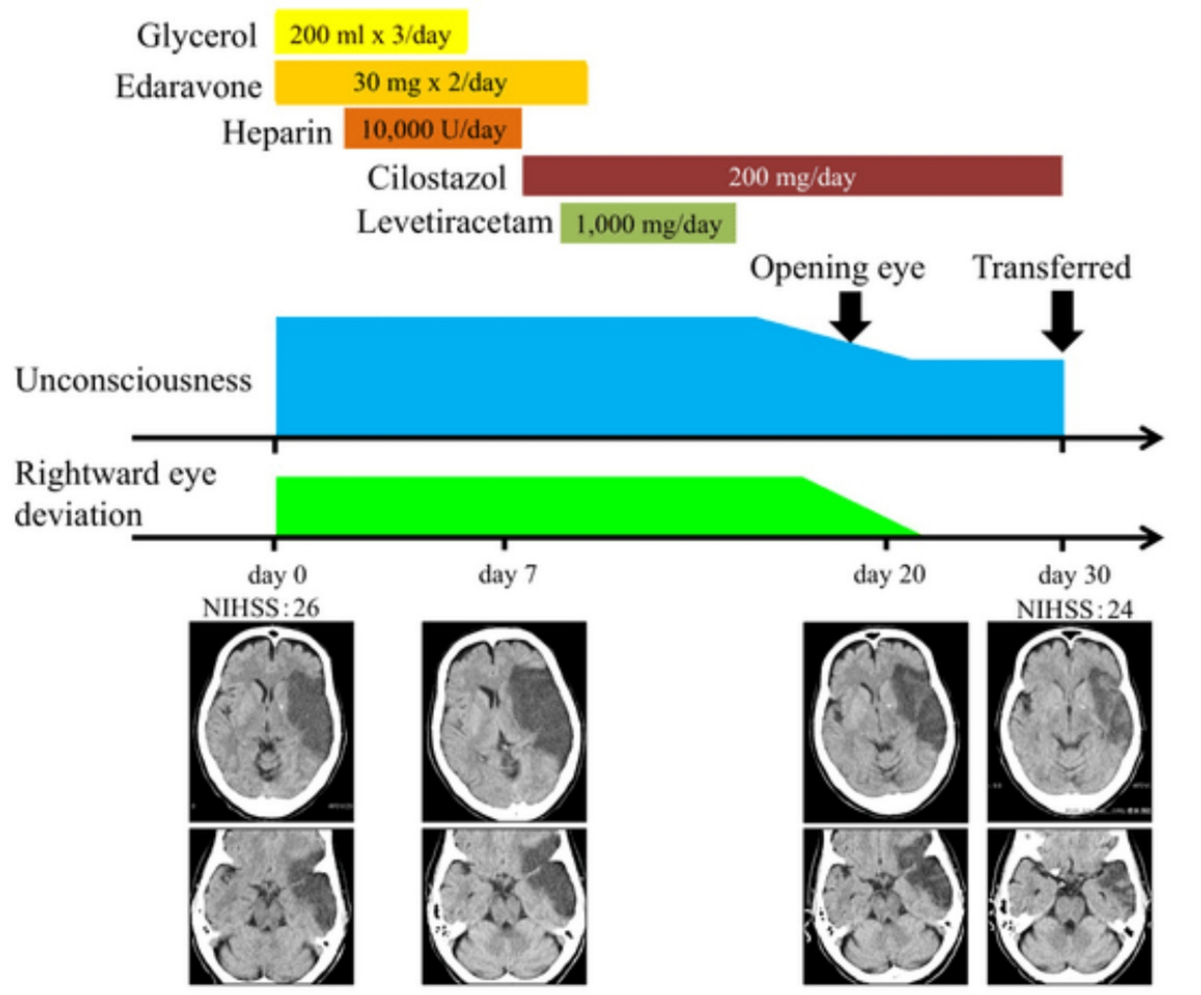
The clinical course of this patient Treatment with edaravone, glycerol, and heparin was started. After the diagnosis of atherothrombotic cerebral infarction based on test results, cilostazol was started. To differentiate epileptic symptoms, levetiracetam was administered, but there was no improvement. Follow-up CT images found no postinfarction hemorrhage or cerebral herniation accompanied by brainstem compression NIHSS: National Institutes of Health Stroke Scale, CT: computed tomography

In the T2* image, the susceptibility vessel sign was absent. The patient was initially considered to have cardiogenic cerebral embolism due to the size of the infarction, and treatment with edaravone, glycerol, and heparin was initiated. Afterward, based on the results of a cervical echocardiogram and Holter electrocardiogram, the patient was diagnosed with atherothrombotic cerebral embolism, and cilostazol was administered. Her electroencephalography (EEG) showed only slow waves from the left hemisphere without epileptic discharges. Considering the possibility of epilepsy, levetiracetam 1,000 mg/day was initiated, but the eye deviation and consciousness disturbance persisted. The eye deviation persisted for about 20 days after admission. Afterward, it gradually disappeared with an improvement in spontaneous eye opening. No abnormalities of pupils' light reflex or anisocoria appeared during the course. The patient's follow-up head CT images found no postinfarction hemorrhage or cerebral herniation accompanied by brainstem compression. One month after admission, the patient was transferred to another hospital. At the time of transfer, her consciousness was GCS 8 (E4V1M3), and her NIHSS was 24 (Figure 3).

## Discussion

**Figure 4:**
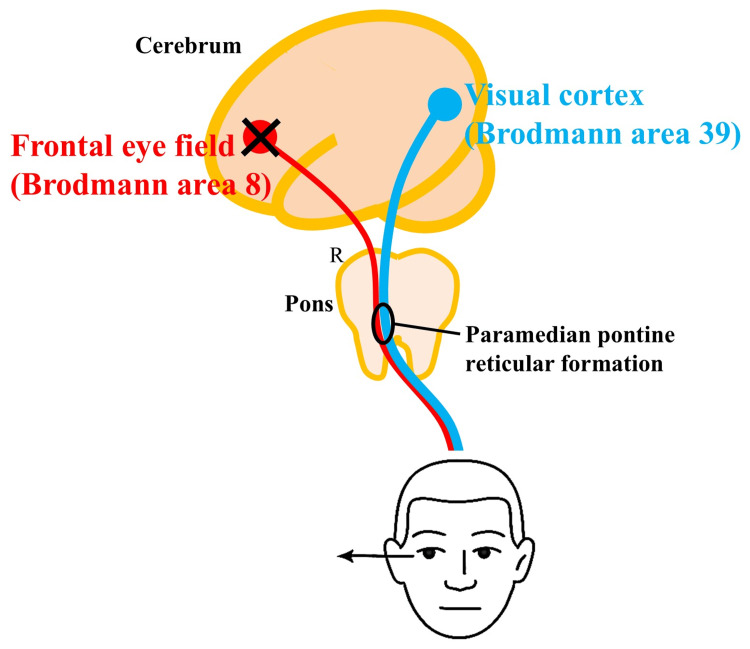
Schematic hypothetical pathway of our patient Our patient’s frontal eye field was involved, but her posterior visual cortex was preserved, which may have resulted in the characteristic “wrong-way eye deviation during eye opening” Image credits: This is an original image created by the author Wataru Shiraishi

It is generally recognized that the supratentorial lesion typically results in eye deviation toward the lesion side, while the infratentorial lesions cause eye deviation toward the unaffected side [1-3]. However, even in supratentorial lesions, eye deviation toward the unaffected, or paralyzed, side may occur, and is called “wrong-way eye deviation” or “contralateral eye deviation" [4]. It is especially common in thalamic hemorrhage [4,6]. But sometimes thalamic infarction also causes wrong-way eye deviation [7]. It has been reported that 1.2% of cerebral infarction patients present with wrong-way eye deviation [4]. The commonly understood mechanism of eye deviation is that damage to one side of the cerebrum causes oculomotor paralysis to the healthy side, resulting in a relative dominance of the contralateral eye rotation function and the appearance of eye deviation toward the affected side [3]. However, the precise mechanism of wrong-way eye deviation is still unclear. So far, three mechanisms are currently postulated: 1) epileptic cerebral activity, especially in thalamic hemorrhage [4,6], 2) contralateral descending oculomotor nerve pathway damage due to brainstem herniation from extensive supratentorial brain lesions [4], and 3) imbalance between the voluntary and automatic oculomotor pathways in the horizontal impulsive eye movement pathway [5,6]. In the present case, the eye deviation was induced only when the eyes were opened forcibly, and the eyes remained in the median position when the eyes were closed. Also, there were no epileptic discharges on EEG, and the antiepileptic drug was not effective for the eye deviation. The involvement of brainstem herniation was also ruled out from the CT examination. In addition, there were no herniation symptoms such as discrepancy of pupils or light reflexes. Therefore, we hypothesized that the imbalance between the voluntary and involuntary oculomotor pathways in horizontal impulsive eye movements may be the hypothesized cause. The hypothesized pathological mechanism is as follows. The horizontal impulsive eye movement pathway is divided into an anterior pathway that controls voluntary eye movement and a posterior pathway that controls automatic eye movement. The anterior tract (in voluntary eye movement) originates from the frontal eye field (Brodmann's area 8) and projects to the contralateral paramedian pontine reticular formation (PPRF), which reactively controls voluntary eye movements [8]. The posterior tract, which controls automatic eye movements, arises from the posterior visual cortex (Brodmann's area 39) and projects to the contralateral PPRF [9]. In the present case, only the anterior tract was damaged by cerebral infarction, but the posterior tract was preserved. The relative dominance of the posterior tract may have resulted in the persistence of a visually guided gaze to the right, resulting in the appearance of co-distraction of the eyes only when the eyes were open (Figure 4).

However, there is no case report of a similar finding among those we could find, and the above discussion is just a hypothesis. Further accumulation of similar cases are necessary.

## Conclusions

Here, we presented a rare case of wrong-way eye deviation in a patient with a massive supratentorial infarction, without brainstem compression or epileptic activity. The deviation occurred only during eye opening, suggesting a unique mechanism involving an imbalance between the voluntary and automatic horizontal eye movement pathways. Specifically, damage to the anterior (voluntary) tract with preservation of the posterior (automatic) tract may have led to visually guided deviation. To our knowledge, no previous reports describe this phenomenon. Further accumulation and analysis of similar cases will be essential to clarify the underlying pathophysiology.
